# Vertebral bone quality score to predict cage subsidence following oblique lumbar interbody fusion

**DOI:** 10.1186/s13018-023-03729-1

**Published:** 2023-03-30

**Authors:** Yong Huang, Qian Chen, Limin Liu, Ganjun Feng

**Affiliations:** 1 Department of Orthopedic Surgery and Orthopedic Research Institute, West China Hospital, Sichuan University, Chengdu, 610041 Sichuan China; 2 Department of Orthopaedics, Affiliated Hospital of North Sichuan Medical College, Nanchong, Sichuan China

**Keywords:** Vertebral bone quality score, Cage subsidence, Oblique lumbar interbody fusion, Risk factors

## Abstract

**Background:**

Current evidence suggests that the magnetic resonance imaging (MRI)-based vertebral bone quality (VBQ) score is a good parameter for evaluating bone quality. We aimed to assess whether the VBQ score can predict the occurrence of postoperative cage subsidence after oblique lumbar interbody fusion (OLIF) surgery.

**Methods:**

Patients (*n* = 102) who had undergone single-level OLIF with a minimal follow-up for 1 year were reviewed in this study. Demographic and radiographic data of these patients were collected. Cage subsidence was defined as ≥ 2 mm of cage migration into the inferior endplate, superior endplate, or both. Further, the MRI-based VBQ score was measured on T1-weighted images. Moreover, univariable and multivariable binary logistic regression analyses were performed. Meanwhile, Pearson analysis was used to evaluate the correlation among the VBQ score, average lumbar dual-energy X-ray absorptiometry (DEXA) T-score, and degree of cage subsidence. Furthermore, ad-hoc analysis was used along with receiver operating characteristic curve analysis to assess the predictive ability of the VBQ score and average lumbar DEXA T-score.

**Results:**

Of 102 participants, cage subsidence was observed in 39 (38.24%) patients. According to the univariable analysis, patients with subsidence had older age, higher antiosteoporotic drug use, larger disk height change, a more concave morphology of inferior and superior endplates, higher VBQ score, and lower average lumbar DEXA T-score compared to patients without subsidence. In the multivariable logistic regression analysis, a higher VBQ score was significantly associated with an increased risk of subsidence (OR = 23.158 ± 0.849, 95% CI 4.381–122.399, *p* < 0.001), and it was the only significant and independent predictor of subsidence after OLIF. Moreover, the VBQ score was moderately correlated with the average lumbar DEXA T-score (*r *= − 0.576, *p* < 0.001) and the amount of cage subsidence (*r* = 0.649, *p* < 0.001). Furthermore, this score significantly predicted cage subsidence with an accuracy of 83.9%.

**Conclusions:**

The VBQ score can independently predict postoperative cage subsidence in patients undergoing OLIF surgery.

## Background

In the literature, many studies have reported favorable clinical outcomes of mini-open oblique lateral interbody fusion (OLIF) in patients with lumbar degenerative disk disease [1, 2]. Moreover, it is possible to perform OLIF through the physiological gap between the aorta and psoas major by making a small anterior–lateral skin incision [3]. This method avoids the invasion into the paraspinal musculature, is less invasive, and reduces the risk of injury to the lumbar plexus; in addition, it offers various advantages, such as minimal trauma and speedy recovery [4]. Indirect decompression of the neural elements is achieved using an enlarged cage to distract the narrow intervertebral space and the foramen [5]. Therefore, the degree of cage subsidence may have a greater impact on the clinical outcomes of OLIF than those of other posterior approach techniques where decompression is achieved directly.

Cage subsidence is one of the most commonly reported complications in OLIF, which leads to various compromised clinical outcomes, such as disk/foraminal height loss, column lordosis reduction, neuroforaminal stenosis, and recurrence of radiculopathy. In the relevant literature, low bone mineral density (BMD) has been considered as one of the main risk factors of cage subsidence, and it could be used as one of its predictors [6, 7]. The gold standard for BMD assessment and osteopenia or osteoporosis diagnosis is dual-energy X-ray absorptiometry (DEXA). However, previous studies have confirmed that DEXA cannot help in accurate BMD assessment of patients with lumbar degenerative diseases. Quantitative computed tomography (qCT) could be used as an alternative method for a more accurate assessment of bone quality. However, qCT is not routinely performed because it is expensive [8, 9]. Recently, a novel method of determining MRI-based vertebral bone quality (VBQ) score was proposed for evaluating the quality and fat infiltration of trabecular bone [10]. This score was found to significantly correlate with BMD assessed using the DEXA scan, which may help reduce investigation costs and radiation hazards [11, 12]. Moreover, the VBQ score has been recognized as an independent risk factor for fragility fractures, and it predicts the occurrence of cage subsidence and reoperation after transforaminal lumbar interbody fusion surgery [13, 14].

However, to the best of our knowledge, no studies have evaluated the relationship between the VBQ score and the occurrence of cage subsidence after OLIF surgery to date. Thus, this retrospective study aimed to assess whether the VBQ score can be used to predict the occurrence of cage subsidence after OLIF surgery.

## Methods

### Patients

We retrospectively collected the medical records of patients who underwent OLIF surgery between February 2019 and October 2021 after obtaining approval from our institutional review board. Patients who underwent single-level OLIF surgery with ≥ 1 year of follow-up were included in this study. In contrast, patients with a history of congenital spinal anomalies, those who did not undergo any surgery for degenerative lumbar disease, those with endplate sclerosis on preoperative CT scan, and those with an endplate injury on immediate postoperative CT scan were excluded from the study. All surgical procedures were consecutively performed at our center by one of the two spine fellowship-trained neurosurgeons.

### Surgical procedure

All procedures were performed by the same group of spine surgeons in accordance with the standard guidelines. After intubation with general anesthesia, the patient was placed in the right lateral recumbent position. A 4–6 cm skin incision was made from the anterior to the center of the marked disk. Further, the retroperitoneal space was accessed by blunt dissection of the abdominal muscle. Moreover, a series of dilators was placed to create an anatomically oblique lateral corridor. After the removal of the disk and preparation of the intradiscal plate, a cage of appropriate size (Clydesdale Spine System, Medtronic, or Oracle Systems, Synthes) filled with demineralized bone matrix and artificial bone material was placed in the optimal position. Subsequently, two screws (Medtronic Sofamor Danek USA, Inc.) were inserted into the lateral side of the vertebral body near the endplate and locked with a single rod. Finally, the abdominal muscles and the incised skin were closed.

### Data collection and radiographic assessment

**Fig. 1:**
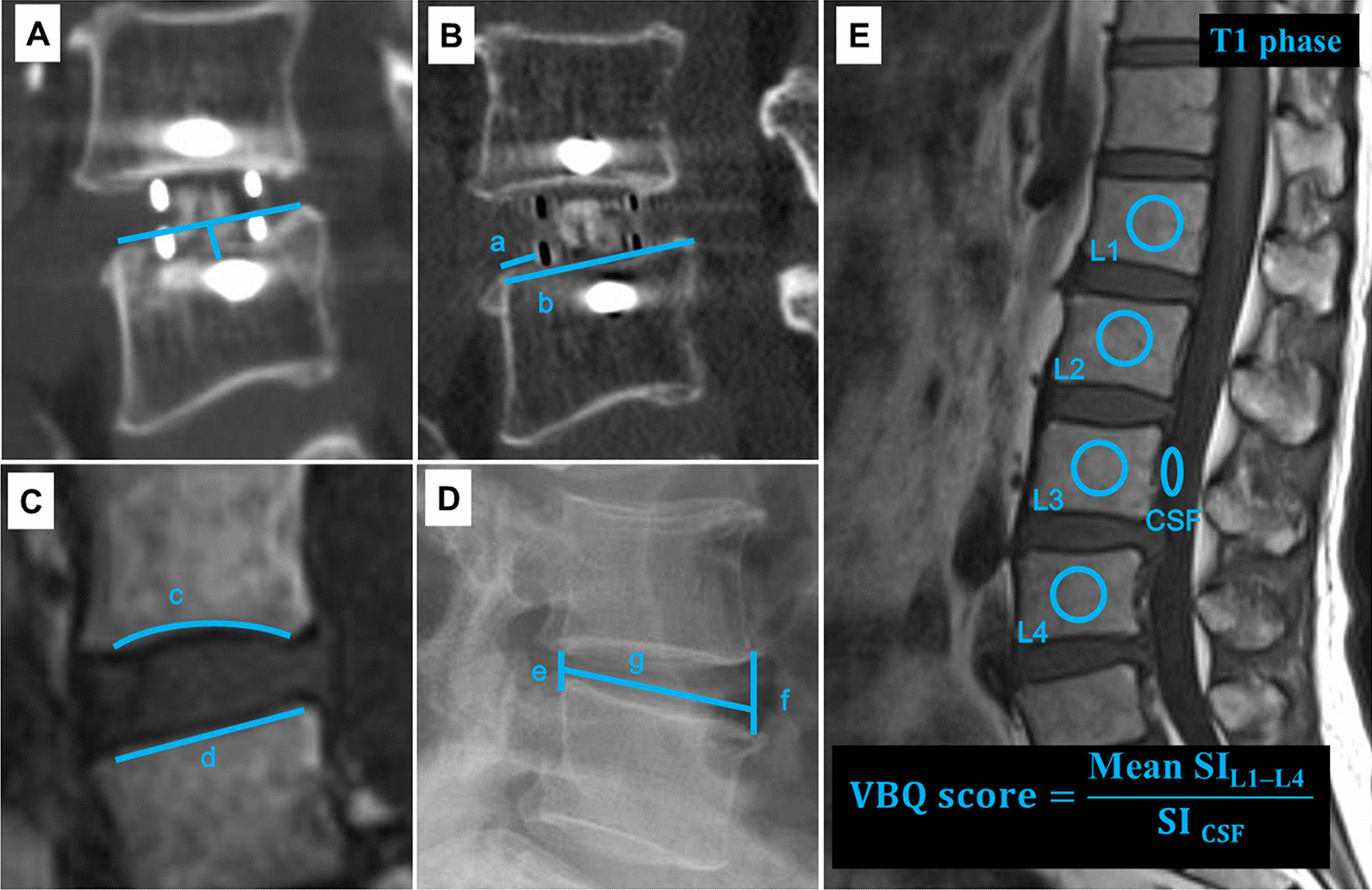
**A** Cage subsidence is indicated as ≥ 2 mm sinking of the cage into the superior endplate. **B** The cage position is calculated as $\frac{a}{b} \times 100\%$ (a, the distance between the anterior metal marker and the leading edge of the caudal endplate; b, he length of caudal endplate). **C** Classification of the disk space morphology on MRI scan (c, concave; d, fat). **D** The disk height is calculated as $\frac{e + f}{g} \times 100\%$ on a lumbar X-ray (lateral view) (e, anterior disk height; f, posterior disk height; g, sagittal disk width). **E** Representative image of the ROI used to calculate the VBQ score

Basic data of the participants, including age, sex, body mass index (BMI), age-adjusted Charlson Comorbidity Index (CCI) score, hypertension, history of smoking and drinking, chronic steroid use, antiosteoporotic drug use for 1 year perioperatively, diagnosis, surgical level, fusion status, and follow-up time, were retrospectively collected. Lumbar CT scans and X-rays of the participants were obtained pre- and postoperatively, i.e., on day 1 and 3, 6, and 12 months postoperatively. We used the average T-scores of lumbar vertebrae 1–4 obtained by dual-energy X-ray absorptiometry (DEXA) scans. Moreover, data related to cage height, length, and position were collected. The primary outcome of this study was cage subsidence, which was defined as ≥ 2 mm of cage migration into the inferior endplate, superior endplate, or both at the final follow-up (Fig. 1A) [15]. Cage position was defined by the percentage of the distance between the anterior metal marker of the cage and the anterior edge of the vertebral body at the length of the caudal endplate [16] (Fig. 1B). The endplate morphology was classified into flat and concave types (Fig. 1C). Disk height was expressed in terms of the Farfan index [13] (Fig. 1D). The change in disk height was defined by the difference between the midline distance of the superior and inferior endplates, and it was assessed before and after 1 day of operation.

### VBQ score calculation

The VBQ score was calculated using the method described by Ehresman et al. [10]. Moreover, a midsagittal T1-weighted MRI of the lumbar spine was performed without inversion recovery or contrast. Regions of interest (ROIs) were placed in the medullary portions of L1–4 vertebral bodies and in the cerebrospinal fluid (CSF) space at the level of L3 (Fig. 1E). Subsequently, the VBQ score was calculated by dividing the mean signal intensity (SI) value in the vertebral bodies of L1–4 by the signal intensity in the cerebrospinal fluid.

### Statistics

Continuous variables were analyzed using the independent *t*-test, whereas categorical variables were compared using the chi-square test. We included all clinically and radiologically significant risk factors in the multivariable logistic regression model. Moreover, Pearson’s correlation test was performed to assess the relationship among the VBQ score, cage subsidence, and lumbar T-score. A receiver operating characteristic (ROC) curve was used for ad-hoc analysis to identify cutoff values by the Youden index and calculate the area under the curve (AUC) of the significant continuous variables. SPSS statistical software version 20 (IBM Corp.) was used to perform statistical analyses. A *p*-value of < 0.05 was considered statistically significant.

## Results

In total, 102 patients were included in this study based on the inclusion and exclusion criteria. The demographic and clinical data of these patients are shown in Table 1. Overall, 39 (38.24%) patients with cage subsidence were included in the subsidence group, and their average subsidence was 3.59 ± 1.22 mm. The remaining 63 patients (61.76%) were included in the nonsubsidence group, and their average subsidence was 1.13 ± 1.56 mm. Comparison between the subsidence and nonsubsidence groups revealed that patients with subsidence were significantly older (62.10 ± 9.60 vs. 58.09 ± 8.82 years, *p* = 0.034) and had undergone more number of antiosteoporotic drug treatments [10 (25.64%) vs. 6 (9.52%), *p* = 0.030] than patients without subsidence. Additionally, patients with subsidence had a larger disk height change (3.51 ± 1.14 vs. 2.99 ± 1.05 mm, *p* = 0.022), more concave morphology of inferior endplate [1 (1.56%) vs. 14 (22.22%), *p* = 0.006] and superior endplate [2 (5.13%) vs. 16 (25.40%), *p* = 0.009] higher VBQ score (3.83 ± 0.82 vs. 2.98 ± 0.39 mm, *p* < 0.001), and a lower average lumbar DEXA T-score (− 1.76 ± 0.98 vs. − 1.02 ± 1.08 mm, *p* = 0.001) than patients without subsidence. In contrast, there were no significant differences in terms of other factors, including sex, BMI, age-adjusted CCI score, hypertension, history of smoking and drinking, chronic use of steroid, diagnosis, follow-up time, surgical level, cage height, cage length, cage position, preoperative and immediate postoperative disk height, and fusion status.

**Table 1:** Comparison of clinical characteristics, surgical factors, and radiographic parameters of the Subsidence and Non-subsidence groups

	Subsidence group	Non-subsidence group	*p*-value
No. of patients	39	63	
Clinical characteristics			
Age (years)	62.10 ± 9.60	58.09 ± 8.82	**0.034**
Sex (male/female)	17/22	32/31	0.479
BMI (kg/m^2^)	25.11 ± 2.97	25.03 ± 3.07	0.895
Age-adjusted CCI	2.15 ± 1.29	1.67 ± 1.34	0.074
Hypertension (Yes/No)	11/28	21/42	0.588
Smoking history (Yes/No)	2/37	9/54	0.147
Drinking history (Yes/No)	4/35	11/52	0.318
Glucocorticoid use > 6 months	2/37	7/56	0.301
Diagnosis			0.131
Lumbar spondylolisthesis	22	43	
Lumbar instability	16	15	
Disk herniation	1	5	
Antiosteoporotic drug treatment (Yes/No)	10/29	6/57	**0.030**
Follow-up time (months)	20.03 ± 4.12	20.10 ± 4.31	
Surgical factors			
Level			0.700
L2–3	2	2	
L3–4	8	17	
L4–5	29	34	
Cage height (mm)	10.77 ± 1.27	10.73 ± 1.61	0.898
Cage length (mm)	48.85 ± 2.68	49.29 ± 3.46	0.500
Cage position	22.72 ± 7.69	24.56 ± 6.76	0.209
Radiographic parameters			
Preop disk height in %	44.26 ± 11.83	48.01 ± 11.66	0.121
Immediate postop disk height in %	64.04 ± 9.66	64.09 ± 9.95	0.979
Change in disk height (mm)	3.51 ± 1.14	2.99 ± 1.05	**0.022**
Superior endplate morphology (Flat/Concave)	2/37	16/47	**0.009**
Inferior endplate morphology (Flat/Concave)	1/38	14/49	**0.006**
Fusion at final follow-up (Yes/No)	8/31	11/52	0.700
Cage subsidence at follow-up(mm)	3.59 ± 1.22	1.13 ± 0.56	**0.000**
VBQ score	3.83 ± 0.82	2.98 ± 0.39	**0.000**
Average lumbar DEXA T-score	− 1.76 ± 0.98	− 1.02 ± 1.08	**0.001**

Bold text denotes statistical significance

All statistically significant factors according to the univariable analysis were included in the multivariable logistic regression model. Detailed results of the effect size of each variable in the multivariable analysis are presented in Table 2. Notably, a higher VBQ score [odds ratio (OR): 23.158 ± 0.849, 95% confidence interval (CI) 4.381–122.399, *p* < 0.001] was the only variable that significantly predicted subsidence. We also conducted correlation analyses to evaluate the correlation among the VBQ score, average lumbar DEXA T-score, and amount of cage subsidence; the results of these analyses are shown in Table 3. A moderate correlation was found between the VBQ score and the amount of cage subsidence (*r* = 0.649, *p* < 0.001). Similarly, the VBQ score was moderately correlated with the average lumbar DEXA T-score (*r* = − 0.576, *p* < 0.001). However, a fair correlation was found between the average lumbar DEXA T-score and the amount of cage subsidence (*r* = − 0.418, *p* < 0.001).

**Table 2:** Results of multivariable analysis: association with increased cage subsidence

Factor	OR	95%CI	*p* Value
VBQ score	23.158 ± 0.849	4.381–122.399	**0.000**
T-score of lumbar spine	1.362 ± 0.354	0.681–2.724	0.382
Antiosteoporotic treatment	2.564 ± 0.915	0.427–15.399	0.303
Age (years)	1.058 ± 0.031	0.995–1.126	0.071
Change of disk height	1.494 ± 0.260	0.897–2.489	0.260
Superior endplate morphology (Flat/Concave)	1.383 ± 1.300	0.108–17.679	0.803
Inferior endplate morphology (Flat/Concave)	0.115 ± 1.688	0.804–3.145	0.200

Bold text denotes statistical significance

**Table 3:** Correlation between the VBQ score, the average lumbar DEXA T-score, and the amount of cage subsidence

Parameter	Correlation coefficient (r)	Degree of correlation	*p* Value
VBQ score cage subsidence	0.649	Moderate	**0.000**
VBQ score average lumbar DEXA T-score	− 0.576	Moderate	**0.000**
Cage subsidence Average lumbar DEXA T-score	− 0.418	Fair	**0.000**

Bold text denotes statistical significance

**Fig. 2:**
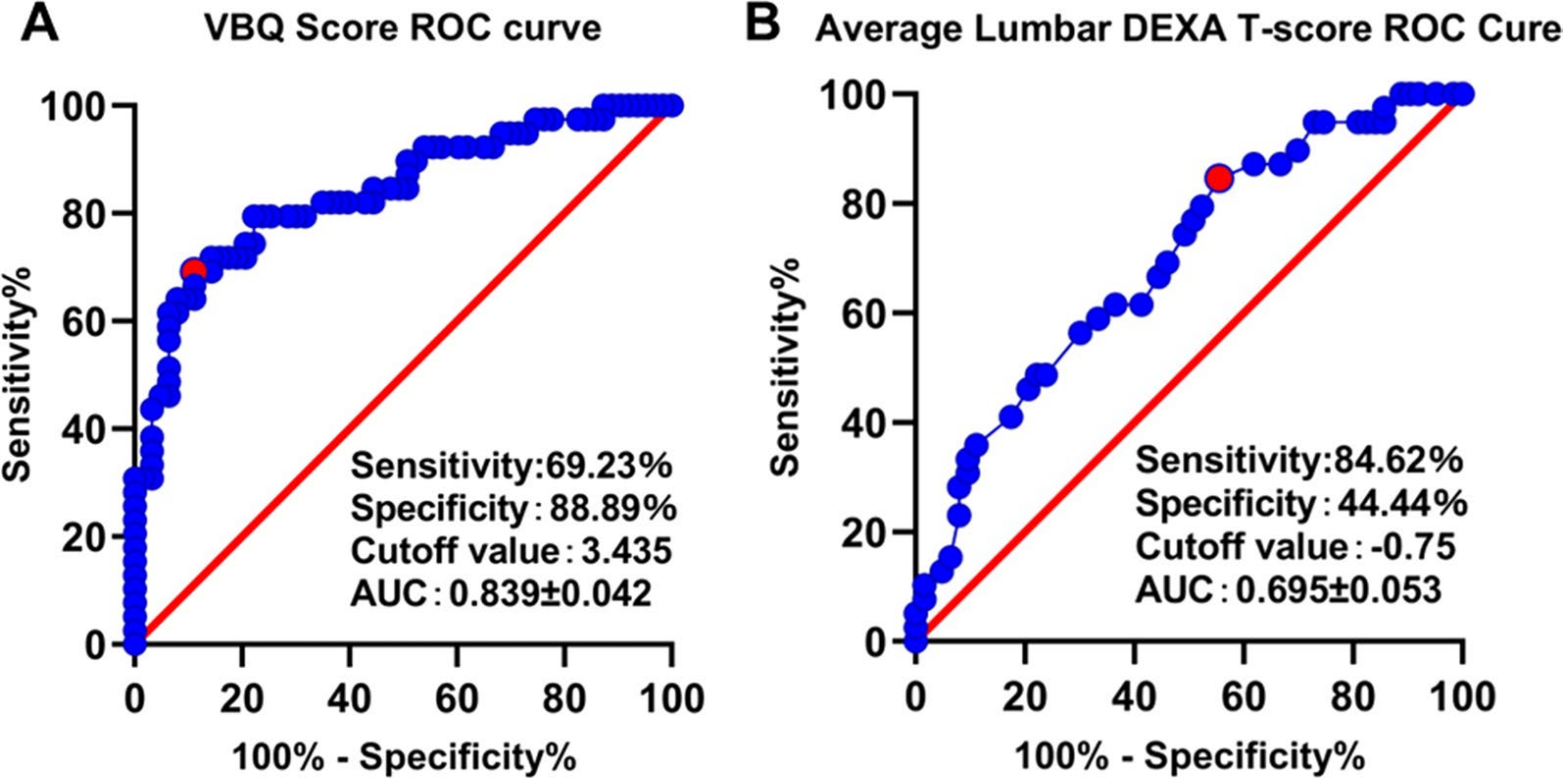
ROC curve analysis and AUC of the VBQ score (**A**) and average lumbar DEXA T-score (**B**) for predicting cage subsidence. Red dots indicate the cutoff points determined by the Youden index

Further, the ad-hoc analysis was performed to determine the cutoff values of the VBQ score and average lumbar DEXA T-score. Moreover, ROC curves were created for the preoperative VBQ score and average lumbar DEXA T-score as predictors for cage subsidence. Notably, the AUCs of the VBQ score and average lumbar DEXA T-score were 0.839 (95% CI 0.756–0.922) and 0.695 (95% CI 0.591–0.798), respectively. The cutoff value of the VBQ score was 3.435, with a sensitivity of 69.23% and a specificity of 88.89% (Fig. 2A). The cutoff value of the average lumbar DEXA T-score was − 0.75, with a sensitivity of 84.62% and a specificity of 44.44% (Fig. 2B).

## Discussion

In the present study, we retrospectively examined 102 patients who underwent OLIF surgery and identified 39 cases of cage subsidence. Some risk factors have been identified including old age, large number of antiosteoporotic drug treatments, increased disk height change, concave endplate morphology, low lumbar DEXA T-score, and high VBQ score. Notably, the novelty of this study is that we found that the VBQ score can independently predict postoperative cage subsidence in patients undergoing OLIF surgery. Cage subsidence is a clinical phenomenon that manifests as sinking of the cage into the vertebrae through an adjacent endplate. This phenomenon may lead to progressive disk height narrowing and poor clinical outcomes. Various subsidence rates have been reported in previous studies, and they range from 11 to 46.7% [3, 5, 17, 18]. In the present study, cage subsidence following OLIF was observed in 38.24% of the participants. This relatively higher subsidence rate in our study can be attributed to the use of a low threshold (≥ 2 mm of cage migration into adjacent endplates) to define subsidence. Moreover, the incidence of subsidence in the present study (38.24%) was much lower than that reported by Kotheeranurak et al. (46.7%), probably because we excluded patients with intraoperative endplate violations [18]. According to the reports in the relevant literature, the probability of occurrence of intraoperative endplate violations ranges from 16.8 to 33.1%, and these violations increase the risk of cage subsidence [19–21]. Therefore, careful management of the surgical procedure is crucial to ensure that the endplate is completely undamaged.

Apart from the endplate violation, the risk factors that were thought to be associated with cage subsidence have been widely discussed in previous studies, including poor bone quality, over distraction, cage position and design, endplate sclerosis, age, and the presence or absence of the supplemental fixation [20, 22, 23]. In the present study, based on univariable analysis, patients with subsidence had older age, underwent greater number of antiosteoporotic drug treatments, had greater disk height change, and had a more concave endplate morphology compared to those without subsidence. Notably, bone quality and muscle mass tend to reduce with aging. According to a previous study, older patients are more likely to develop cage subsidence owing to the negative effects of age on bone microstructure [24]. Moreover, the use of antiosteoporotic drugs was recommended postoperatively for patients undergoing lumbar fusion surgery in order to reduce the subsidence rate [25]. However, in our study, the number of patients treated with antiosteoporosis therapy was more in the subsidence group, indicating that such patients have worse bone quality and are more prone to cage subsidence. Over the distraction is another important factor contributing to cage subsidence. Notably, it has been reported that disk space distraction may cause cage subsidence in anterior cervical and lumbar fusion cases [26, 27]. A large distraction leads to a large change in disk height, which further causes a significant increase in the compressive force between the cage–endplate interface. The selection of cage height has been suggested to be determined according to the disk height measured preoperatively. Furthermore, previous reports have proven that cage morphology affects cage subsidence [28, 29]. In particular, it has been believed that a flat endplate has better interface contact with an OLIF cage at the surface. A well-matched endplate-cage surface provides more even stress distribution and a larger area for endplate coverage, decreasing the incidence of cage subsidence. In contrast, a concave endplate provides a reduced contact area and leads to stress concentration, increasing the incidence of cage subsidence [30].

Low BMD has been proven to be a critical risk factor for cage subsidence. Proper preoperative assessment of bone quality helps in developing a reasonable surgical plan and taking relevant preventive measures to improve surgical outcomes and reduce associated complications. Although DEXA is recognized as the gold standard for bone density assessment, it is associated with many issues that can lead to inaccurate evaluations, such as spinal deformities, overlying soft tissue, previous compression fractures, bowel content, osteophytes, and aortic atherosclerosis [31–33]. In the present study, the average lumbar DEXA T-score was fairly correlated with the amount of cage subsidence; this finding was similar to the findings of previous studies, which reported that BMD assessed by DEXA scanning had a significantly weak correlation with the amount of cage subsidence. Therefore, this score is not a good predictor for cage subsidence in OLIF surgery and demonstrates low accuracy (0.695). Previous studies have reported that Hounsfield units calculated by qCT may help assess bone quality more accurately than the DEXA T-score [9, 34]. Notably, low Hounsfield units indicate low BMD, which further indicates a higher incidence of developing cage subsidence [7, 35]. However, qCT is more expensive and associated with a high risk of radiological hazards; hence, it is not suitable for use in routine examinations.

Previous studies have examined the MRI-based VBQ score to assess bone quality [10, 36]. In particular, the use of this score is inexpensive and completely radiation-free. Osteoporotic bone is usually characterized by trabecular atrophy and local adipocyte replacement on histological analysis [37]. The use of the VBQ score is based on the theory that more atrophy and fatty infiltration in osteoporotic trabecular bone causes higher SI on T1-weighted images. Owing to the easy-to-teach methodology and rapid results unaffected by confounding factors, the measurement of this score has shown good reproducibility and reliability, demonstrating that this is a practical tool for clinical use [14, 38]. In previous studies, the use of the VBQ score was verified against the use of DEXA to identify osteoporotic bone and to independently predict the risk of fragility fractures, cage subsidence, and vertebral compression fracture in patients with spine metastases [13, 39, 40]. In the present study, the VBQ score was moderately correlated with the average lumbar DEXA T-score and amount of cage subsidence. This score is known to be an accurate indicator of bone quality; in addition, it is known to facilitate the identification of osteoporotic bone. More importantly, the VBQ score was found to be a significant independent predictor of cage subsidence after OLIF surgery according to the multivariable logistic regression analysis in our study. Patients with VBQ scores higher than the threshold are approximately 23 times more likely to experience subsidence than those with lower scores. Moreover, the VBQ score predicts cage subsidence better than the average lumbar DEXA T-score, with an accuracy of 0.839. This difference may be attributed to the fact that the VBQ score is a site-specific bone density measure that directly reflects vertebral bone quality at the weight-bearing site while excluding factors affecting whole-region standard DEXA measurement, making its assessment more precise than that of the DEXA T-score. Notably, endplate bone quality—a novel site-specific MRI-based bone quality assessment proposed by Jone et al. [41] could predict severe cage subsidence after standalone lateral lumbar interbody fusion even better than the VBQ score. These findings suggest that the VBQ score should be adopted in clinical practice for patients with OLIF surgery in order to improve the efficacy and safety of treatment.

This study has several limitations. First, the retrospective design was subject to certain biases, such as the selection bias in the data collection and the potential observer-expectancy bias. Second, the relatively small cohort from a single health center may explain the lack of statistical significance of some previously identified risk factors for cage subsidence in our multivariable logistic regression analysis. Finally, some important laboratory examination parameters were not assessed in the present study, including calcium, phosphorus, lipids, and cholesterol levels. A previous study reported that patient physiology affects the VBQ score and found a higher VBQ score in patients with hyperlipidemia and healthy bones [42].

## Conclusion

The VBQ scoring method can be used to evaluate bone quality with good effectiveness and accuracy and to independently predict postoperative cage subsidence in patients undergoing OLIF surgery. Preoperative measurement of the VBQ score should be considered to identify high-risk patients and take timely precautions to minimize complications.

## Data Availability

Data will be available upon request to the corresponding author.

## Abbreviations

VBQ: Vertebral bone quality
MRI: Magnetic resonance imaging
OLIF: Oblique lumbar interbody fusion
BMD: Bone mineral density
DEXA: Dual-energy X-ray absorptiometry
CT: Computed tomography
BMI: Body mass index
CCI: Charlson Comorbidity Indexs
ROI: Regions of interest
CSF: Cerebrospinal fluid
SI: Signal intensity
ROC: Receiver operating characteristic
AUC: Area under the curve
